# Oxidation of Sperm DNA and Male Infertility

**DOI:** 10.3390/antiox10010097

**Published:** 2021-01-12

**Authors:** Leila Rashki Ghaleno, AliReza Alizadeh, Joël R. Drevet, Abdolhossein Shahverdi, Mojtaba Rezazadeh Valojerdi

**Affiliations:** 1 Department of Embryology, Reproductive Biomedicine Research Center, Royan Institute for Reproductive Biomedicine, ACECR, Tehran 16635-148, Iran; Lrashki@yahoo.com (L.R.G.); alizadehmasouleh@royaninstitute.org (A.A.); shahverdi@royaninstitute.org (A.S.); 2 Faculty of Medicine, GReD Institute, INSERM U1103—CNRS UMR6293—Université Clermont Auvergne, CRBC Building, 28 Place Henri Dunant, 63001 Clermont-Ferrand, France; 3 Department of Anatomy, Faculty of Medical Science, Tarbiat Modares University, Tehran 14155-6343, Iran

**Keywords:** spermatozoa, oxidative DNA/nuclear damage, DNA fragmentation, epigenetic alteration

## Abstract

One important reason for male infertility is oxidative stress and its destructive effects on sperm structures and functions. The particular composition of the sperm membrane, rich in polyunsaturated fatty acids, and the easy access of sperm DNA to oxidative damage due to sperm cell specific cytologic and metabolic features (no cytoplasm left and cells unable to mount stress responses) make it the cell type in metazoans most susceptible to oxidative damage. In particular, oxidative damage to the spermatozoa genome is an important issue and a cause of male infertility, usually associated with single- or double-strand paternal DNA breaks. Various methods of detecting sperm DNA fragmentation have become important diagnostic tools in the prognosis of male infertility and such assays are available in research laboratories and andrology clinics. However, to date, there is not a clear consensus in the community as to their respective prognostic value. Nevertheless, it is important to understand that the effects of oxidative stress on the sperm genome go well beyond DNA fragmentation alone. Oxidation of paternal DNA bases, particularly guanine and adenosine residues, the most sensitive residues to oxidative alteration, is the starting point for DNA damage in spermatozoa but is also a danger for the integrity of the embryo genetic material independently of sperm DNA fragmentation. Due to the lack of a spermatozoa DNA repair system and, if the egg is unable to correct the sperm oxidized bases, the risk of de novo mutation transmission to the embryo exists. These will be carried on to every cell of the future individual and its progeny. Thus, in addition to affecting the viability of the pregnancy itself, oxidation of the DNA bases in sperm could be associated with the development of conditions in young and future adults. Despite these important issues, sperm DNA base oxidation has not attracted much interest among clinicians due to the lack of simple, reliable, rapid and consensual methods of assessing this type of damage to the paternal genome. In addition to these technical issues, another reason explaining why the measurement of sperm DNA oxidation is not included in male fertility is likely to be due to the lack of strong evidence for its role in pregnancy outcome. It is, however, becoming clear that the assessment of DNA base oxidation could improve the efficiency of assisted reproductive technologies and provide important information on embryonic developmental failures and pathologies encountered in the offspring. The objective of this work is to review relevant research that has been carried out in the field of sperm DNA base oxidation and its associated genetic and epigenetic consequences.

## 1. Introduction

The increasing prevalence of infertility in couples is a worldwide health problem affecting roughly 15% of couples. Infertility is defined as the inability of a couple to conceive after at least one year of unprotected intercourse. According to WHO statistics, the inability to procreate has been increasing in recent years, and many couples of childbearing age suffer from this problem [1]. Numerous studies have shown that women and men share equal responsibility in that situation, i.e., about 30% to 40% for each partner [2], the remaining share representing cases for which the origin of infertility cannot be attributed to one or the other partner.

Fragmentation of sperm DNA, whether single-stranded or double-stranded, is the most recognized form of DNA damage to the sperm nucleus. High sperm nucleus fragmentation has been clearly associated with an increased risk of miscarriage, poor embryonic quality, and implantation failure [3]. These last years, sperm DNA fragmentation (SDF) has been the subject of intense study, and there appears today to be a consensus on the fact that the degree of sperm nucleus fragmentation is a reliable indicator of reproductive success, although there is not yet a consensus regarding the technique to be used to diagnose it.

A long-lived misconception that generates a lot of confusion in both the scientific and clinical communities is the thinking that SDF is a read-out of sperm DNA oxidative alterations. In fact, SDF is partly attributable to oxidative stress as it can also result from unresolved meiotic breaks, the remnant of apoptotic cells not fully evacuated during spermatogenesis, or mechanical breaks in the sperm nucleus especially upon chromatin remodeling and histone to protamine replacement at the onset of spermiogenesis. When oxidative stress is important SDF is most often associated, however, sperm DNA oxidation is a condition that can occur alone (i.e., not associated with SDF) and must be considered on its own.

Sperm DNA oxidative stress studies over the past decade have shown that nucleosides, particularly guanosine and adenosine, are very sensitive to oxidation. In addition, it appears that oxidative stress may also affect the sperm epigenome, with possible adverse effects on embryo development [4]. In this context, the level of oxidation of the spermatic nucleus is a parameter that must be seriously considered both in the development of tools to measure this type of damage and in understanding the consequences it may have. A better knowledge of these aspects could certainly contribute to obtaining improved results in assisted reproduction techniques (ARTs) for couples concerned with male factor-related infertility. This review aims to bring into focus the importance of sperm DNA oxidation in male infertility and its possible developmental and transgenerational consequences.

## 2. Reactive Oxygen Species and Male Infertility

**Figure 1:**
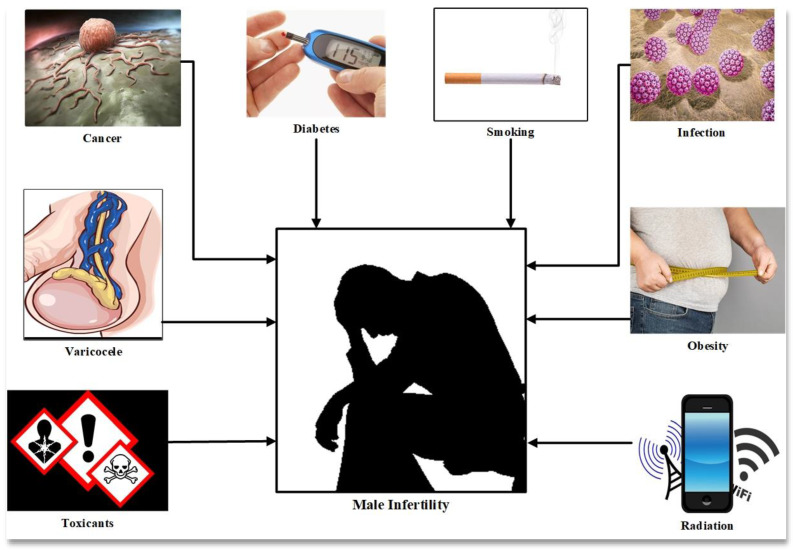
The causative factors in male infertility.

**Figure 2:**
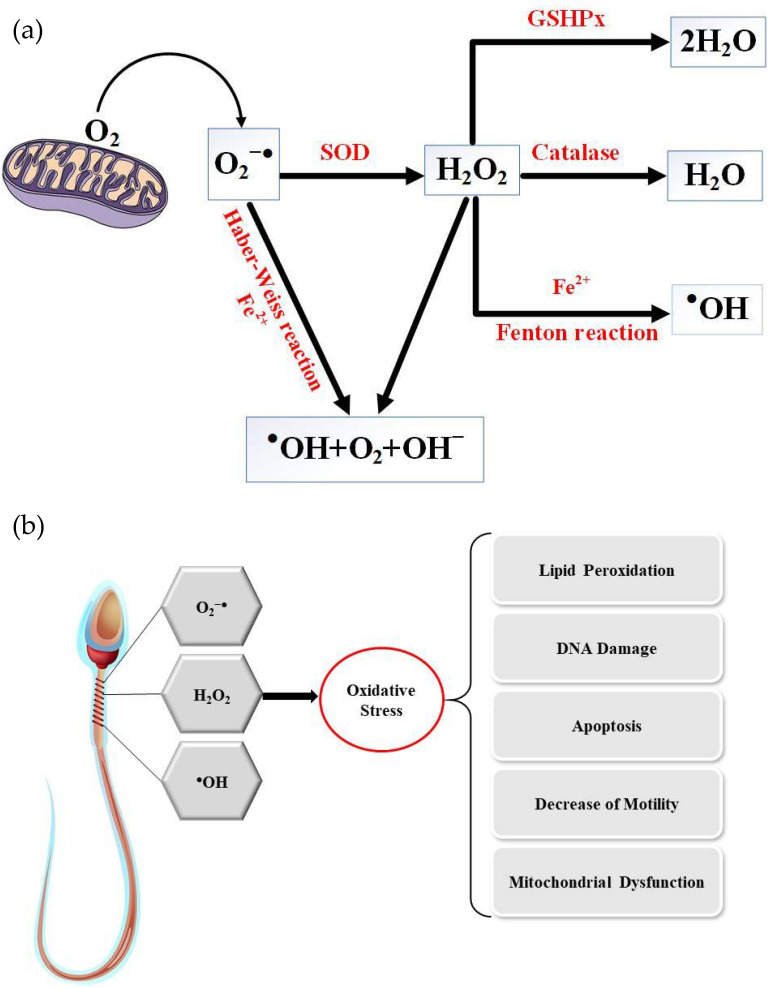
(**a**) Reactive oxygen species generation and (**b**) consequences on spermatozoa structure and function. O_2_^●−^: superoxide anion; H_2_O_2_: hydrogen peroxide; ^●^OH: hydroxyl radical; ^●^OH: hydroxide; SOD: superoxide dismutase.

Male infertility is caused by a number of factors such as individual genetics, lifestyle and environmental factors and clinical causes such as varicocele, endocrine imbalance and infection, all of which are associated with oxidative stress and DNA damage (Figure 1) [5]. Oxidative stress (OS) is mediated by reactive oxygen species, molecules inherent to the consumption of oxygen by the cells of aerobic organisms. Cellular respiration, which provides the energy to support cell metabolism and life for aerobic organisms, consumes oxygen and inexorably generates active oxygen derivatives (free radicals and activated oxygen molecules/reactive oxygen species (ROS)). The main sources of ROS are intracellular at the level of the mitochondrial respiratory chain, which converts oxygen into ATP, and also during enzymatic reactions of glycolysis in the cell cytosol. The univalent production cascade of ROS starts with the dismutation of oxygen giving rise to the superoxide anion radical (O_2_^●–^), which is recycled by superoxide dismutase (SOD) into a non-radical ROS, hydrogen peroxide (H_2_O_2_). Unlike the superoxide anion, which remains close to its source of production and has a very short life (in the range of nanoseconds), hydrogen peroxide is very stable and could cross the plasma membrane and contact all cellular and extracellular compartments. Via the Fenton and Haber–Weiss reactions, an excess of H_2_O_2_ leads to the generation of very aggressive free radicals (hydroxyl and alkoxyl radicals, ^●^OH and OH^–^). In addition, peroxidation of unsaturated fatty acids in lipids, the target of choice of ROS, gives birth to lipid hydroperoxides and aldehydes which are very aggressive to cell components, often perpetuating the generation of more ROS (Figure 2) [6].

It is intriguing to note that the evolution of pluricellular animals has not seen fit to equip cells with specific enzymatic activities to recycle the very aggressive free radicals (^●^OH and OH^−^) resulting from H_2_O_2_ excess. These molecules, if they are in excess, will attack all cellular constituents and eventually lead to cell death. The metazoan evolutionary choice has been to finely control the intracellular and extracellular H_2_O_2_ concentrations through a battery of cytosolic and/or secreted enzymes such as catalase, glutathione peroxidases, peroxiredoxins, glutathione-S-transferases and glutaredoxins that turn H_2_O_2_ into something neutral, i.e., water, H_2_O. In addition, several non-enzymatic primary antioxidants are there both in the intra- and extra-cellular compartments to protect cell components from free radical attacks by readily quenching them. These small metabolites include but are not limited to glutathione, thioredoxin, poly-amines, vitamins such as E and C, taurine and hypotaurine [7].

Like all aerobic cells of metazoans, sperm cells are constantly exposed to oxygen (O_2_) and the problems that come with it. In mammals, the spermatozoa, of all the cells that make up tissues and organs, is the most ROS-susceptible cell type. As early as 1943, MacLeod reported that radical attacks due to an oxygen-rich environment resulted in reduced sperm motility [8]. Paradoxically, around the same time, Tosic and Walton (1946) demonstrated that the sperm itself produced large amounts of ROS [9]. A simple search in PubMed using the keywords “oxidative stress” and “spermatozoa” reveals more than 3000 articles devoted to this subject. Despite this intense and long-standing research, many questions concerning the effects of oxidative stress on sperm structures and functions and the role oxidative sperm damage plays in male infertility as well as its consequences for the embryo and offspring still remain without clear answers [10].

Briefly, spermatozoa have unique features making them susceptible to oxidative damage. Mature spermatozoa are silent cells with no capacity to respond to stress stimuli because the haploid and highly compacted nucleus does not transcribe anymore. In addition, non-genomic responses are expected to also be very poor because this cell has evacuated most of its cytoplasm during the last stages of spermatogenesis. The latter also explains why this cell is not well equipped in cytosolic antioxidants that are normally present in the cytosol of any other somatic cell types. In addition, the quasi “silent” nature of spermatozoa explains why this cell is not able to repair its genetic material from oxidative alteration, as the base excision repair (BER) pathway that should take care of oxidized bases (as is the case in any other cell) was shown to be truncated in mammalian spermatozoa, leaving this task to be completed by the oocyte DNA repair machinery after fertilization [11]. Adding up to this already oxidative-stress-susceptible situation, spermatozoa possess a peculiar plasma membrane that is very rich in polyunsaturated fatty acids (PUFAs), targets of choice of ROS, leading to the production of toxic aldehydes themselves embarking the cell in an ROS-generation vicious circle [12]. Any unreasonable increase in lipid peroxidation and disruption of membrane structure leads to loss of sperm motility [13]. These particular features make spermatozoa structure and function very susceptible to oxidative damage and it was with no surprise that oxidative stress was readily shown to be associated with loss of sperm membrane fluidity, mitochondria dysfunction (both affecting sperm motility), impaired gamete recognition, increased abnormal morphology, decreased sperm viability, premature acrosome exocytosis and defective signaling events during capacitation leading to fertilization failures [13,14].

However, the picture got more complicated as spermatozoa were finally shown to have an ambiguous relation with oxidative activities. Firstly, ROS were shown to be part of the signaling cascades leading to spermatozoa activation [15]. Sperm capacitation and acrosome reaction partly depend on ROS, which are necessary for efficient fertilization [15]. It was shown that optimal levels of ROS facilitate the production of intracellular ATP and phosphorylation of axoneme proteins, which enhance sperm motility, while low levels of ROS reduce sperm motility. Secondly, ROS (in particular H_2_O_2_) were also more recently shown to be physiologically necessary to some extent for the post-testicular epididymal maturation of spermatozoa structures [16]. Therefore, ROS are both necessary and dangerous for sperm cells. They are necessary because they act as second messengers in oxygen-dependent signal transduction pathways and because they enable physiologically required oxidative processes (e.g., disulfide bridging of protein thiol residues), which can take place either spontaneously or via the action of disulfide isomerases in the presence of H_2_O_2_ in both cases. However, when accumulated in excess, activated oxygen species attack all organic components (lipids, carbohydrates and proteins, including nucleic acids) that can lead to cell death. ROS are therefore somehow the proverbial double-edged sword; on the one hand, they are beneficial for some physiological activities and, on the other hand, an excess of ROS is detrimental to cell structures, functions, and survival [10,17].

Being both beneficial and detrimental to spermatozoa structures and functions implies that ROS have to be finely regulated around spermatozoa. Since spermatozoa are poorly autonomous for their protection they thus rely on their environment (the epididymal fluid during post-testicular maturation and storage and, subsequently, the seminal fluid upon ejaculation) to protect themselves from oxidative damage. That is why the fluids surrounding spermatozoa were found to contain a wide range of both non-enzymatic and enzymatic molecules having antioxidant properties [18]. It is interesting to note at this stage that if spermatozoa structure and function are susceptible to oxidative alterations, it does not necessarily mean that the more antioxidant protection you have the better the situation is. Spermatozoa also fear reductive conditions [19,20], implying that a very finely tuned balance should exist between oxidative processes and antioxidant capacities.

## 3. The Unique Organization of the Sperm Nucleus Explains Its ROS Susceptibility

**Figure 3:**
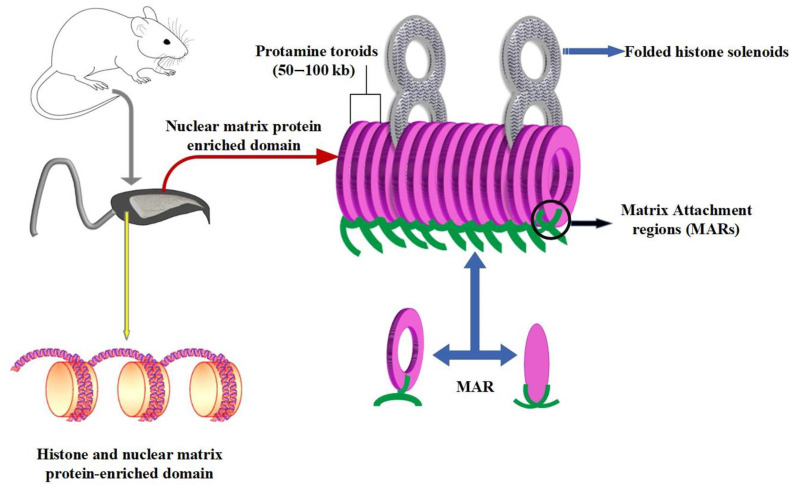
In the mouse sperm nucleus, there is a higher protamine content in the center; while persisting histones are enriched in peripherical and basal head domains in close association with nuclear matrix proteins (modified from [32]).

During spermatogenesis, prior to meiosis, the chromatin of differentiating male germ cells is in its organization similar to that of somatic cells with chromatin embedded in nucleosomes around testicular histone octamers (TH1, TH2A, TH2B and TH3) [21]. After meiosis and during the spermiogenesis process, new DNA binding proteins (transition proteins, TPs) will sequentially replace the histones and allow a change in the organization of the spermatic chromatin. Finally, in the final stages of the spermatid transformation protamines will replace TPs. Protamines are rich in cysteine, arginine (55 to 79%), lysine and histidine. They are very basic and smaller in size than histones (5 to 8 kD) allowing greater compaction of the chromatin [22] via the formation of toroidal structures onto which 50 to 100 kb of DNA is rolled up (compared to 146 bp on an octamer of histones in somatic-type nucleosomes). The family of protamines includes two members, protamine type 1 (P1) and protamine type 2 (P2). P1 is found in all mammalian species, while P2 is found only in the sperm nucleus of some mammalian species such as humans, mice and horses [23,24]. A unique aspect of the chromatin structure of mammalian spermatozoa is therefore its level of compaction, which allows a decrease in the size of the sperm nucleus, which is thus six to seven times smaller than a somatic cell nucleus [25,26]. This decrease in the size of the sperm nucleus is furthermore enhanced during epididymal transit by inter-and intra-protamine disulfide bridging of the thiol groups carried by the numerous protamine cysteine residues [27]. In this particular assembly of the paternal genetic material, it should be noted that only 90 to 95% of the sperm DNA is combined with protamines. The remaining fraction of the spermatic chromatin remains in a nucleosomal assembly associated with paternal histones [28,29]. The distribution of these residual histones is not random and, according to reported data, they are distributed in two regions of the spermatic chromatin/nucleus. Some of these persistent spermatic nucleosomes are found in large regions (about 10 kb) called “solenoids” that interrupt the toroidal ring stacks of the sperm chromosomes. Nucleosomes are also found in the short strands of DNA (about 1 kb in length) that link each toroid to its neighbors (Figure 3). These linker regions are themselves associated with the protein nuclear matrix and contain the origins of paternal DNA replication. They constitute the first structures of the paternal nucleus to respond to the oocyte environment after fertilization. Due to their lower compaction and their association with the peripheral nuclear matrix, making them easily accessible to extrinsic stressors such as ROS, these nucleosomal regions are particularly susceptible to oxidative damage [30,31]. 

## 4. Sperm DNA Fragmentation versus Sperm DNA Oxidation

Many investigators, both clinical and scientific, agree on the relationship between oxidative stress and sperm DNA fragmentation. A simple search on the NCBI website using the keywords “Spermatozoa & Oxidative Stress” yields 3051 articles dealing specifically with the fragmentation of sperm DNA following oxidative stress. Studies conducted worldwide have shown that single- or double-strand breaks of the sperm genome beyond a certain threshold have adverse effects on fertility in both natural conception and ARTs [33,34]. Existing literature clearly shows that sperm DNA fragmentation is increased in physiological and/or pathophysiological situations such as aging, inflammation and infections [35,36]. In addition, environmental factors such as occupational stress, unbalanced diet, exposure to chemical toxicants, physical stresses such as extreme heat or cold, and exposure to ionizing or non-ionizing radiation have been directly linked to high sperm DNA damage and increased percentage of immature sperm, largely due to the pro-oxidative aspects of these diverse and potentially cumulative situations [37].

While it is clear that part of the fragmentation of sperm DNA has an oxidative origin, it should be remembered that other reasons may explain this situation. Indeed, fragmented spermatic DNA may be due to unrepaired meiotic breaks, poor evacuation of apoptotic germ cells during spermatogenesis or/and necrotic cells during epididymal maturation, or mechanical breaks in the spermatic nucleus upon spermiogenesis during the replacement of histones by protamines [38]. Thus, while a pro-oxidative situation may lead to sperm nucleus fragmentation, it should not be considered as synonymous with sperm DNA oxidation alone. On the other hand, and equally if not more relevant, the absence of sperm nucleus fragmentation at a level considered pathological (see the determination of the level of spermatic nucleus fragmentation), should not be interpreted as the absence of sperm DNA oxidation. In fact, it has been shown that oxidation of the bases of sperm DNA is much more frequent than sperm nucleus fragmentation. Studies have shown that in a panel of men from infertile couples, 2 to 3 out of 10 had a level of sperm nucleus fragmentation considered pathological but 6 to 7 out of 10 had a moderately to highly oxidized sperm nucleus [39,40]. The study of Vorilhon et al. showed that the level of oxidation of the sperm nucleus was well correlated with asthenozoospermia and leukocytospermia [39], situations in which oxidative stress is well known to be at stake. This study also showed a positive correlation between male BMI, another clinical situation well known to generate systemic oxidative stress, and the level of oxidation of the spermatic nucleus [39]. In two studies on Chinese men working in coal mines and having infertility issues, an increase in the oxidation of sperm DNA bases and the production of significant amounts of oxyguanine in these individuals following inhalation of carbon dioxide were observed. However, in neither of the two studies did the rate of DNA fragmentation differ significantly between mine workers and the control groups [32,41]. These observations highlight the fact that the study of sperm DNA fragmentation alone does not provide a complete picture of the state of genomic damage.

Thus, since sperm nucleus oxidation does not explain all the fragmentation of sperm DNA, and the absence of fragmentation cannot be an assurance of the absence of oxidation, it is clear that the two parameters (sperm nucleus fragmentation and sperm nucleus oxidation) must be evaluated to properly qualify the state of the sperm nucleus and develop the most appropriate therapeutic strategy [42].

## 5. Sperm DNA Oxidation a Hidden Danger

Since oxidation of the sperm DNA, although it may accompany nucleus fragmentation, can frequently exist independently of it, the question is then, what are the consequences of this situation when it exists alone? This has been clearly demonstrated in transgenic animal models in which epididymal luminal antioxidant protection has been lowered, resulting in a significant increase in the number of oxidized bases on the sperm nucleus independently of any other alterations [43,44]. Indeed, in this model, the oxidation generated was not sufficient to induce sperm nucleus fragmentation and the epididymis of transgenic mice found ways to compensate for sperm membrane lipid peroxidation [44,45]. The sperm nucleus of these transgenic mice was shown to contain a high level of oxidized guanosine residues, the so-called 8-oxodG or 8-OHdG, the most susceptible nucleoside to oxidation [43]. The presence of these oxidized bases was correlated with chromosomal domains still associated with nucleosomes and particularly to those inter-toroid DNA linker strands associated with the sperm nuclear matrix, most likely because of their peripheral nuclear localization [43,46]. It should be noted that if guanosine is the most susceptible to oxidation, adenosine is next and the two other bases (cytosine and thymidine) may get oxidized too, although to a lesser extent. Therefore, by detecting solely 8-OHdG residues one gets only a part of the real picture of the sperm DNA oxidative alteration. Interestingly, not all mouse chromosomes were found to be equally susceptible to DNA oxidation. In the mouse model, a gradient of damage was found with smaller chromosomes being more susceptible to oxidative damage than longer ones [43,46]. The rationale for this was that in the mouse sperm nucleus, small chromosomes are more peripheral than longer ones [43,46]. A parallel situation was found in humans with histone-enriched regions of chromosomes and, again, especially DNA linker strands in nucleosomal organization connecting protamine toroids, being susceptible to oxidation [47]. In contrast with the mouse situation, in the human sperm nucleus, the number of oxidized 8-OHdG bases found on chromosomes followed a linear relation with their respective length [47]. This discrepancy was explained by the fact that the human sperm nucleus is a lot less condensed than the mouse sperm nucleus, authorizing ROS to penetrate deeper inside it, readily contacting each chromosome. This lower level of nuclear compaction of the human sperm nucleus is due to its higher content in persisting histones approximately 10 times more represented than in the mouse sperm nucleus [16,47]. In the mouse and the human, some special hot spots for sperm DNA oxidation were identified [43,47]. It is interesting to note that human chromosome 15 is by far the most sensitive to DNA oxidation in a region known to bear loci involved in syndromes occurring more frequently in children from aging couples [47].

Interestingly, mating transgenic males with an oxidized sperm nucleus with wildtype females of proven fertility revealed that there was no impact on fertilization success rates but that it was associated with impaired developmental processes [44]. In particular, higher miscarriage rates, higher abnormal developments and higher perinatal mortality were recorded when compared to control mating with wild-type males [44]. In humans, hotspots for sperm DNA oxidation were investigated in fertile and infertile cohorts and it was observed that they were significantly more oxidized in infertile males when compared to fertile ones [47].

## 6. Undisputable Consequences of Sperm DNA Oxidation

**Figure 4:**
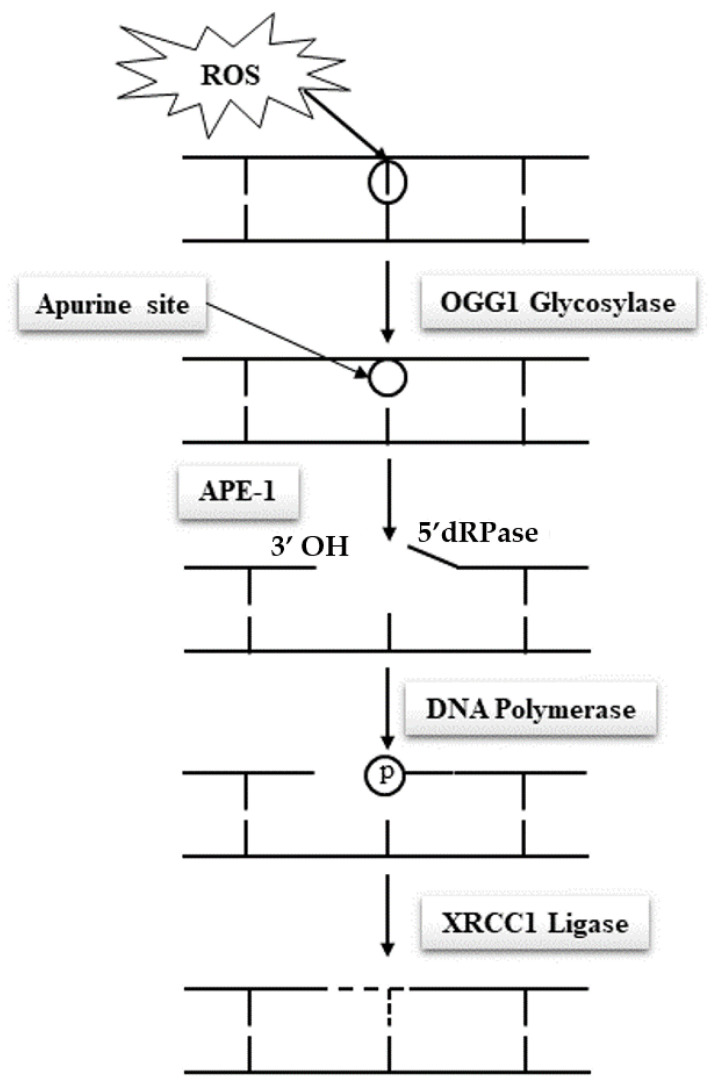
A representation of the base excision repair (BER) pathway. In this pathway, the oxidized bases are identified and removed with the help of the OGG1 glycosylase, and the APE1/XRCC1 recombinase. Spermatozoa possess only the first part of the BER pathway, but lack the APE-1/XRCC1 activities [46]. ROS: reactive oxygen species; APE-1: apyrimidinic/apurinic endonuclease 1; 5′dRPase: 5′-terminal deoxyribose phosphatases; XRCC1: X-ray repair cross-complementing.

Due to their specific features, spermatozoa are quasi-silent cells devoid of most of the functional attributes of any other cell type. In particular and of utmost importance, mature spermatozoa do not have a functional DNA repair system, which makes these cells unable to correct DNA base alterations. Classically, the base excision repair (BER) pathway oversees the removal of all damaged nucleotides as a result of oxidation or deamination. In the case of oxidized guanosine and adenosine residues, by far the most frequent situations due to the susceptibility of these bases to oxidation, the BER system involves the activity of the 8-oxoguanine glycosylase (OGG1), which cuts off the oxidized guanine so-called 8-oxo-guanine or 8-hydroxyguanine (8-oxo-7,8-dihydroguanine = 8-OHdG) creating an open apyrimidine site (AP site). Next, the apyrimidinic/apurinic endonuclease 1 (APE1), together with the scaffolding protein known as X-ray repair cross-complementing (XRCC1) that interacts with DNA ligase and polymerases, completes the BER pathway and fills in the AP site with an unaltered residue (Figure 4). Unfortunately, spermatozoa have been shown to lack a complete BER system. They only possess OGG1 activity but lack the APE1 and XRCC1 activities [48]. Oxidized guanine residues in the sperm DNA will thus be readily processed by the sperm OGG1, creating AP sites that will be carried out in the oocyte upon fertilization. It will be the task of the maternal APE1 and XRCC1 activities to complete the BER pathway in the hours that follow fertilization during the complex events leading the sperm nucleus back to a somatic like configuration allowing the fusion of the parental genetic materials. This gender shared process is a nice example of the complementary roles played by the two gametes in the process of generating a new individual. It also brings forward the major role played by the oocyte in repairing the paternal genetic material.

Many situations may affect the efficacy by which the oocyte BER pathway works. Fertilization with spermatozoa bearing a high level of oxidized residues that will potentially overwhelm the oocyte repair capacity is one of these situations. On the female side, oocytes coming from aging women or oocytes that have been stressed following for example hormonal stimulation during ART, chemo or radiation therapies or cryoconservation, all situations that are known to reduce egg quality, represent other classical cases that could lead to incomplete sperm nuclear repair, potentially leading to adverse developmental consequences including an increased mutational load in the next generation [49,50,51,52,53,54]. Any weakness in the spermatozoa’s ability to create abasic sites via its OGG1 activity when facing excessive oxidation of guanine residues, in addition to any weakness in the oocyte ability to complete the BER pathway after fertilization, will therefore promote a classical Hoogsteen-type base pair mismatch, which will lead to de novo G to T transversion mutations that will be carried on to any cell of the developing embryo [16,55].

It is interesting to note that 80% of the 40,000 or so de novo mutations (not present in any of the parents) one records in offspring are of paternal origin, with most of them being of the transversion type. This observation underlines the very important role of the paternal DNA in the transmission of genetic alterations potentially involved in increasing miscarriage rates and infertility, abnormal embryonic development, and the increased susceptibility of the next generations to disease. It is also interesting to note that the number of mutations attributable to sperm increases by 1.51 per year with the father’s age, while the number of mutations attributable to oocyte increases by 0.37 mutations per year with the mother’s age, again emphasizing the paternal share in the offspring mutational load [56].

In this context, one understands how important the criteria of gamete quality and sperm nuclear integrity are. This should prompt the clinical community to a more critical perception of our current approach of evaluating the male partner of infertile couples. Despite undisputable data associating sperm DNA/nuclear loss of integrity, among which sperm DNA oxidation is by far the most common, with reproductive failures there is still no routine evaluation of the integrity of the paternal nucleus and DNA. Considering the dual role of oxidation on spermatozoa being both detrimental when in excess and serving physiological processes when optimal, several parameters should be evaluated to get a precise view of the sperm nuclear oxidative status. In our opinion a complete view of the sperm nuclear oxidative status could be given by the monitoring of three distinct parameters: sperm nuclear condensation, sperm nuclear fragmentation and sperm DNA oxidation.

## 7. Sperm Nuclear Oxidation and Epigenetic Alteration: More to Be Afraid of

There are obvious reasons to suspect that spermatozoa nuclear oxidative damage goes beyond its impact on the DNA bases and the level of DNA fragmentation.

Oxidative stress has been clearly associated with global sperm DNA hypomethylation, which in turn was associated with loss of chromatin integrity and infertility [57,58,59]. One explanation was that the presence of 8-OHdG within CpG islands could affect the methylation of the adjacent cytosine leading to hypomethylation [60].

Besides interfering with the efficiency of the DNA methylation process, it is likely that oxidative stress may also change the ratio between methyl cytosine (mC) and hydroxymethylcytosine (hmC), another mark associated with mC that is today considered to carry on itself genuine epigenetic information. The simple result of the oxidation of mC is hmC. It is classically generated through the action of the ten eleven translocase (TET) enzymatic family but can occur spontaneously in a pro-oxidative situation. TET-mediated formation of hmC is the first step towards the erasure of the mC mark post-fertilization during the particularly active demethylation process of the male pronucleus. If most of the male pronucleus DNA will be demethylated, there are however regions of the paternal nucleus that will remain methylated at paternally imprinted genes and genomic regions in which transposons must be maintained silent. Preliminary data in animal models suggest that in the situation of post-testicular oxidative stress, one assists in an increase in the level of hmC on the sperm nucleus. Depending on where these changes occur, if it concerns regions that should not be engaged in the demethylation process post-fertilization, it may have some unforeseen consequences on the developing embryo and future individual. As an example, it is suspected that autism, a highly prevalent child disorder associated with paternal age, is linked to sperm DNA methylation errors secondary to oxidative alterations [61,62,63].

Besides DNA methylation, histone modification, and in particular histone methylation, constitutes another epigenetic mark that could be modified in the situation of oxidative stress. To our knowledge, to date there are no data showing that sperm persisting histones might be differentially methylated/hydroxymethylated in the situation of oxidative stress, whether it is testicular and/or post-testicular. However, since sperm persisting histones are transferred to the zygote upon fertilization and are not replaced by oocyte-contained histones, one cannot exclude that, if oxidized, they could interfere with developmental processes associated with the sperm chromatin regions concerned.

A third epigenetic mark associated with the spermatozoa is more and more convincingly suspected to be modified in the situation of oxidative stress. This concerns the complex load of small non-coding RNAs (ncRNA) that accompanies mature spermatozoa and is delivered to the oocyte upon fertilization. It was shown these last years that part of these sperm ncRNAs is of epididymal origin and that it was very dynamic in reflecting paternal pathophysiological status. It was recently reported that, in an animal model of post-testicular oxidative stress, the epididymal epithelial ncRNA profile was significantly changed, especially when it came to the PiR (PiwiRNA), MiR (microRNA) and tRNA-derived small RNA families [64]. It comes as no surprise that, since the epididymis epithelium was shown to transfer proteins and RNAs to transiting spermatozoa via apocrine secretion, preliminary investigations have revealed that the spermatozoa ncRNA content in this animal model of post-testicular oxidative stress was modified when compared to control animals (C. Chu, unpublished data).

Although we are far from understanding all the fine modifications generated by oxidative stress to the epigenetic information carried by spermatozoa, there are already convincing shreds of evidence that the epigenetic profile of spermatozoa is changed when challenged by oxidative conditions. Whether these discrete changes will have consequences for the zygote, the embryo, the next generation and beyond remains to be evaluated.

## 8. The Pertinence of Antioxidant Therapeutic Strategies

Whether antioxidant (AO) supplementation could be a valuable therapeutic approach for the infertile male/couple is at the center of a very disputed debate. Up to now, and unfortunately for the field, despite many attempts, the question has never been properly addressed in a well-designed clinical trial. The major issues lie with inappropriate patient selection protocols, inappropriate choice of antioxidant(s) used, inappropriate dose administration, the pertinence of the endpoints monitored and low-powered cohorts, to cite just a few. This highlights the frequent problems associated with current published reports, rendering interpretation of results meaningless. The last of the kind, the so-called MOXI trial, is yet another example of an ill-designed trial [65], rendering arbitrary conclusions that are likely to occult the benefits of AO supplementation when it is correctly handled. A published reflection on this article is however correct in its title, stating, “is it time to stop routinely recommending antioxidant therapy to infertile men?” Our answer is yes, only those patients presenting sperm DNA oxidative damage should be treated, and this implies that they should be evaluated prior to any antioxidant treatment. In our opinion, it is not logical to offer treatment to men with poor semen parameters or sperm DNA damage, rather than specifically to men with seminal oxidative stress. Antioxidants will only protect those sperm subjected to oxidative stress, which was not even assessed in the MOXI study. In addition, it should be pointed out that poor semen parameters and sperm DNA fragmentation can be caused by a multiplicity of factors independent of oxidative stress, which are not going to be helped by antioxidant supplements.

It comes as no surprise that, knowing that oxidative processes are, on the one hand, necessary to achieve optimal sperm structures and function, and on the other hand, detrimental to sperm structures and function when in excess, the subject is complex and cannot be answered by outright judgments. Because of this duality, antioxidants should not be given to anyone without knowing whether it is necessary to supplement or not. Monitoring sperm oxidative damage should then be the appropriate way to go, whether it is seminal ROS content, sperm membranous lipid-peroxidation or sperm DNA oxidation, the latter being in our opinion the best way to go as, as we have seen previously, sperm DNA/nuclear oxidation is the starting point of many problems that may show up during embryonic development and/or in the next generation. Direct assessment of sperm DNA oxidative damage is, in our opinion, an important tool, as we have already discussed the point that if sperm DNA fragmentation may have an oxidative origin, the absence of a pathological sperm DNA fragmentation level does not ensure that there is no sperm DNA oxidation. Identically, monitoring sperm nuclear condensation as an indirect way to evaluate oxidative sperm DNA damage may be correct in some cases but non-appropriate in other cases, as DNA breaks that are not due to oxidative stress may explain the suboptimal condensation of the sperm nucleus. One limitation, explaining why the evaluation of sperm DNA oxidation is not routinely performed in ART clinics, comes from the fact that the technologies require some technical expertise as they use either immunofluorescence microscopy, flow cytometry or HPLC [38,41,66]. In addition, these techniques have a non-negligible cost and are time-consuming. When automatized by flow cytometry, which is the most accurate way to go as it prevents operator bias, it is not appropriate for patients with very low sperm counts (severe oligozoospermia).

Over-supplementation or supplementation of patients having no oxidative stress issue brings the opposite risk of reductive stress that is as detrimental to the sperm nucleus integrity as oxidative stress. As oxidative processes participate in the optimal achievement of sperm structures, excess of AOs may logically be detrimental. AO excess could likely limit the condensation of the sperm nucleus by preventing the formation of disulfide bridges between protamines normally occurring during epididymal maturation, consequently making the sperm nucleus less resistant to aggressors and more prone to get damaged. Therefore, it is essential to adjust the AO treatment in terms of dose, duration and molecules used to the level of oxidative damage recorded on the sperm nucleus/DNA of each individual.

## 9. Conclusions

Sperm DNA/nuclear oxidative damage appears to be a major threat, in particular in terms of reproductive success, as it may have multiple impacts on the developing embryo and on the progeny. This review has emphasized the fact that sperm DNA/nuclear oxidation should not be considered synonymous with sperm DNA/nuclear fragmentation. If in some cases sperm DNA fragmentation can be a consequence of heavy sperm DNA oxidation, moderate/medium sperm DNA oxidation is frequently not associated with DNA fragmentation. Therefore, evaluation of sperm DNA/nuclear integrity should not be done solely via the monitoring of sperm DNA/nuclear fragmentation. Direct evaluation of sperm DNA oxidative damage should complete the assessment of the infertile male. Only when this parameter is evaluated will it be possible to (1) properly assess its impact on embryo development and pregnancy outcome as, to date, data are limited; (2) properly assess the pertinence of oral antioxidant supplementation as an adequate therapeutic strategy. Another point that should be evaluated further is to what extent ART procedures are themselves the source of sperm DNA/nuclear oxidative damage, which may explain their limited success rate. In addition, with regard to the potential modification of sperm genetic and epigenetic marks mediated by oxidative stress, the question of whether or not ART procedures introduce adverse unforeseen issues in the progeny and beyond should be evaluated.
